# A Genetic Multimutation Model of Autism Spectrum Disorder Fits Disparate Twin Concordance Data from the USA and Canada

**DOI:** 10.1155/2015/519828

**Published:** 2015-03-04

**Authors:** Ivan Kramer, Paul H. Lipkin, Alison R. Marvin, Paul A. Law

**Affiliations:** ^1^Physics Department, University of Maryland Baltimore County, 1000 Hilltop Circle, Baltimore, MD 21250, USA; ^2^Johns Hopkins School of Medicine, Baltimore, MD 21205, USA; ^3^Department of Medical Informatics, Kennedy Krieger Institute, 3825 Greenspring Avenue, Baltimore, MD 21211, USA; ^4^Universite Protestante au Congo, Kinshasa 2, Democratic Republic of the Congo; ^5^Johns Hopkins School of Public Health, Baltimore, MD 21205, USA

## Abstract

Whether autism spectrum disorder (ASD) is caused by genetics, environmental factors, or a combination of both is still being debated today. To help resolve this issue, a genetic multimutation model of ASD development was applied to a wide variety of age-of-onset data from the USA and Canada, and the model is shown to fit all the data. Included in this analysis is new, updated data from the Interactive Autism Network (IAN) of the Kennedy Krieger Institute in Baltimore, Maryland. We find that the age-of-onset distribution for males and females is identical, suggesting that ASD may be an autosomal disorder. The ASD monozygote concordance rate in twin data predicted by the genetic multimutation model is shown to be compatible with the observed rates. If ASD is caused entirely by genetics, then the ASD concordance rate of a cohort of monozygote twins should approach 100% as the *youngest* pair of twins in the cohort passes 10 years of age, a prediction that constitutes a critical test of the genetic hypothesis. Thus, by measuring the ASD concordance rate as a cohort of monozygote twins age, the hypothesis that this disorder is caused entirely by genetic mutations can be tested.

## 1. Introduction

Autism Spectrum Disorder (ASD) develops early in life in susceptible children and affects social communication, interaction skills, and behavior. The definition of ASD used by the American Psychiatric Association (APA) has changed over the years, with the current diagnostic criteria for the disorder described in the Diagnostic and Statistical Manual, Fifth Edition (DSM-5). Although the relative contributions of environment and genetics in causing ASD have long been debated, early twin studies suggest that genetics is a major contributor. The goal of this paper is to construct a genetically driven, multimutation model for ASD development and test the model using monozygote and dizygote twin pair data where one member of each pair has developed the disorder. This paper applies the successful modeling of schizophrenia by Kramer and Hong 2013 [1] to ASD. A complete description of the model appears in the appendices.

## 2. Modeling ASD Twin Concordance Data

The new age-of-onset data in this paper comes from the Interactive Autism Network (IAN) database established by the Kennedy Krieger Institute in Baltimore as of September 22, 2014 (to be called the 2014 IAN database herein). A comprehensive treatment of ASD twin data conducted by Rosenberg et al. [2] was based on an earlier version of this database. Of the 277 twin pairs in the Rosenberg study, 59 out of the 67 monozygote twin pairs (88.1%) and 64 out of the 210 dizygote twins (30.5%) were found to be pairwise concordant. Instead of publishing the ASD age of first diagnosis distribution for this cohort of twins, Rosenberg et al. published the mean age (37.9 months) and standard deviation (20.4 months) at first diagnosis instead. The mean age and standard deviation for all twin pairs in the 2009 study turned out to be 7.7 years and 3.5 years, respectively.

New cases were added to the IAN database since 2009, and some of the older twin pairs were omitted in this analysis because of incomplete questionnaires, yielding a new total of 320 twin pairs; however, these omitted pairs still exist in the IAN database. The differences in the number of monozygote and dizygote concordant twins in the IAN database used in Rosenberg et al. [2] and the corresponding number used in the present analysis is almost entirely due to different inclusion criteria.

There were a total of 518 twin cases (123 monozygotic twins with a concordance of 77.2% and 395 dizygotic twins with a concordance of 27.8%) in the original data set. A disproportionate number of* concordant* twin data (for both monozygote and dizygote twins) was missing the age-of-diagnosis of the cotwin, and, therefore, did not meet a necessary requirement to be included in this analysis. This led to a final data set with 90 monozygotic twins (68.3% concordant) and 230 dizygotic twins (18.9% concordant). Concordant twins were less likely to be included in the data set because of the extra work required of the parents completing forms. For an unaffected twin to be included in the data set no additional forms are required beyond basic registration. Another way of saying this is that concordant twins required twice as much effort on behalf of the parent than did nonconcordant twins. This tendency causes a bias that works against the core hypothesis of this work.

In the current IAN study, the average age and standard deviation at ASD diagnosis are 41.2 months and 25.2 months, respectively, slightly higher than what they were in the 2009 IAN study.

The population of Maryland is 5.9 million, 1.8% of the population of the USA of 316 million. There are only 16 twin sets out of 320 from Maryland, representing 5% of our total sample. Although there are 10 more Maryland cases in our database than expected, it is not sufficient to substantially bias the sample. We feel that the IAN database used in this analysis is a reasonable representation of the USA as a whole.

**Figure 1:**
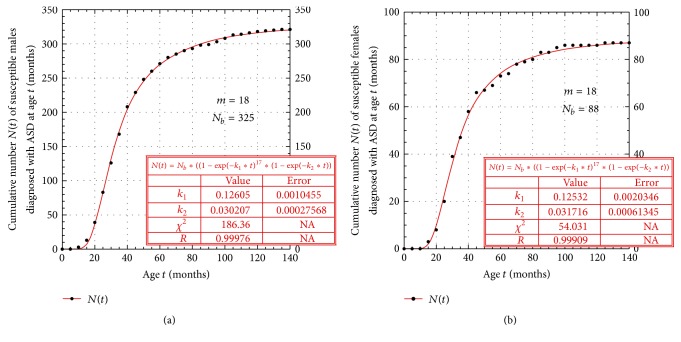
(a) Male ASD age-of-onset *N*(*t*) data and model fit for *N*_*b*_ males born with the susceptibility to develop the disorder. (b) Female ASD age-of-onset *N*(*t*) data and model fit for *N*_*b*_ females born with the susceptibility to develop the disorder.

The cumulative number of* males N*(*t*) who were officially diagnosed with ASD as a function of age *t* in the 2014 IAN database is plotted in Figure 1(a). This data was fitted with the model function, obtained from (A.5) in Appendix A, given by

(1) Nt=Nb·1−exp⁡⁡−k1tm11−exp⁡⁡−k2t≡Nb·Pst,

where *N*_*b*_ is a fourth parameter to be determined by the fit. Clearly *N*_*b*_ is the total number of males in this database that are susceptible to developing ASD in a lifetime. The results of the fit are also shown in Figure 1(a), and the values of the intrinsic model parameters are reproduced in Table 1.

Similarly, the cumulative number of* females N*(*t*) who were officially diagnosed with ASD as a function of age *t* in the 2014 IAN database is plotted in Figure 1(b). Similarly fitting this data with the function in (1) yields the analogous results in Figure 1(b) and Table 1. Comparing the corresponding male and female intrinsic parameter results in Table 1 shows that the values of the corresponding model fit parameters are practically identical to each other! The immediate conclusion of this result is that ASD develops in exactly the same way in males and females, suggesting that ASD may be an autosomal disorder.

Humans inherit 23 chromosome pairs from their biological parents, one set of 23 from their fathers and another set of 23 from their mothers. These chromosomes are identified by a numbering scheme with labels 1 through 23 with the chromosome that determines the sex of the child deliberately placed last (number 23). The sex chromosome comes in two types, called X and Y. Males inherit an X and a Y chromosome, while females inherit two Xs. The chromosomes 1 through 22 are called autosomes. Genes found on one of the 22 autosomal pairs (not on an X or Y sex chromosome) are called autosomal genes, they also come in pairs, and mutations in these genes lead to* autosomal* disorders. If only* one* copy of a gene needs to be defective (mutated) to cause a disorder, the disorder is said to be* dominant*. If* both* copies of a gene need to be defective to cause the disorder, the disorder is said to be* recessive*.

There is evidence in the literature that autism is an autosomal disorder (see, e.g., Ritvo et al. [3] and Barrett et al. [4]). Interestingly, Barrett et al. [4] found mutations associated with autism on the autosomal chromosomes (1–22) with the strongest results for chromosome 13 and 7.

In our modeling of the male and female ASD age-of-onset curves, we found that both curves were convincingly described by the same function with the same parameters. Since the male and female ASD age-of-onset curves appear to be identical, the development of this disorder appears to be independent of the sex of the patient in this dataset. Thus, our modeling suggests that autism is not only an autosomal disorder, as suggested by Ritvo et al. [3] and Barrett et al. [4], but also ASD, the full spectrum of these disorders, as well. In the modeling that follows we will assume that the male values of the intrinsic parameters in Table 1 apply to both males and females.

The evidence that genetics plays an important role in the development of ASD is contained in a recent study of the medical records of all children born in Sweden between 1982 and 2006 [5]. A total of 14,516 children were identified with ASD out of a total of just over 2 million screened, the largest study of its kind. The researchers found that the closer an individual is genetically to a relative with ASD, the greater the risk the individual has of also acquiring ASD. So, for example, siblings born to parents that already had a child with ASD were found to have a 10.3 times greater risk than normal of developing the disorder. It remains to determine whether or not environmental factors also contribute to ASD development. Perhaps the definitive answer to this question can come from follow-up studies of ASD monozygote twin concordance data, as we shall soon show.

In the 2014 IAN database used in this study about four times as many males are born with the susceptibility to develop ASD as do females, a typical disparity in ASD studies. A wide variety of different hypotheses have been advanced to explain this gender disparity in ASD prevalence. The most recent such hypothesis comes from a comparative study of copy-number variants (CNVs) of a particular gene and single-nucleotide DNA sequence variants (SNVs) in males and females with ASD [6]. The researchers found that females with ASD had a significantly higher number of both CNVs and SNVs than males with ASD, suggesting that it is harder for females to develop this disorder than males. However, our modeling fits to male and female age-of-onset ASD data shows that both curves are* identical*, with the same number of mutations required to trigger the full onset of the disorder. Thus, it is possible that the excess number of variants seen in females with ASD compared to males with ASD may have little or nothing to do with causing the disorder.

An alternative possible explanation for the gender disparity in ASD prevalence is male gender bias, that is, females are simply less likely to be diagnosed with the disorder. Remember there is no biological test that can objectively identify ASD in an individual, only a checklist of behavioral symptoms characteristic of the disorder. Because of inherent differences in male and female personalities, it is possible that many female ASD cases simply get overlooked and undiagnosed. This possible explanation for ASD gender disparity is compatible with our modeling results, namely, that the age-of-onset curves for males and females are identical.

**Figure 2:**
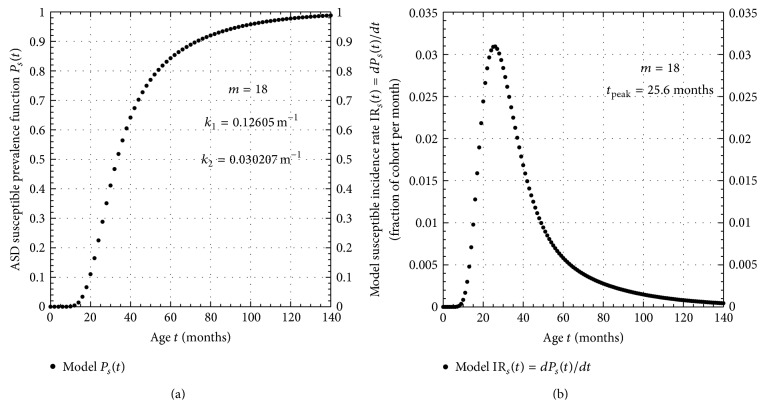
(a) Model susceptible prevalence function *P*_*s*_(*t*) obtained by fit to ASD data. (b) Plot of model incidence rate IR_*s*_(*t*) obtained from fit to ASD age-of-onset data for a cohort susceptible to developing the disorder.

A plot of the susceptible prevalence curve *P*_*s*_(*t*) obtained by the model fit to the 2014 IAN data is shown in Figure 2(a). The *P*_*s*_(*t*) curve predicts that 50% of those born with a susceptibility to develop ASD will develop it by the age of 33 months (2.75 years), while 98% will develop it by the age of 124 months (10.3 years). A plot of the slope of the *P*_*s*_(*t*) curve, IR_*s*_(*t*) = *dP*_*s*_(*t*)/*dt*, is contained in Figure 2(b) and shows that the susceptible incidence rate curve IR_*s*_(*t*) peaks at the age of 25.6 months (2.13 years). Note that the total area under the complete IR_*s*_(*t*) curve must equal 1.

**Figure 3:**
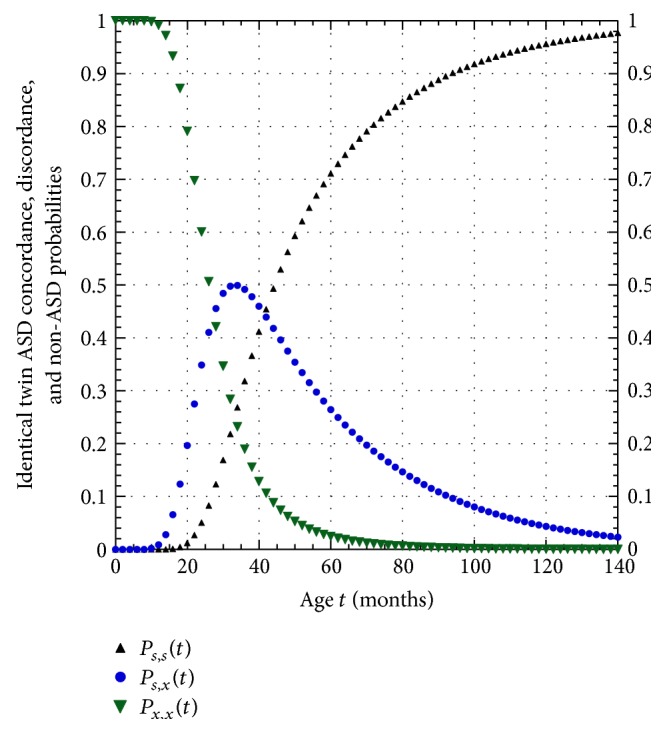
Plots of ASD concordance probability *P*_*s*,*s*_(*t*), discordance probability *P*_*s*,*x*_(*t*), and non-ASD probability *P*_*x*,*x*_(*t*) for identical twins susceptible for developing ASD using the model *P*_*s*_(*t*) curve.

Figure 3 contains plots of the three functions given in (B.2) in Appendix B for the concordance, discordance, and non-ASD probability functions for identical twins predicted by the model fit to IAN data. Thus, as seen in Figure 3, given a cohort of identical twin pairs born with the susceptibility to develop ASD, by the age of 40 months, 41.2% are concordant, 46.0% are discordant, and in 12.8% of the pairs neither twin has yet developed the disorder.

**Figure 4:**
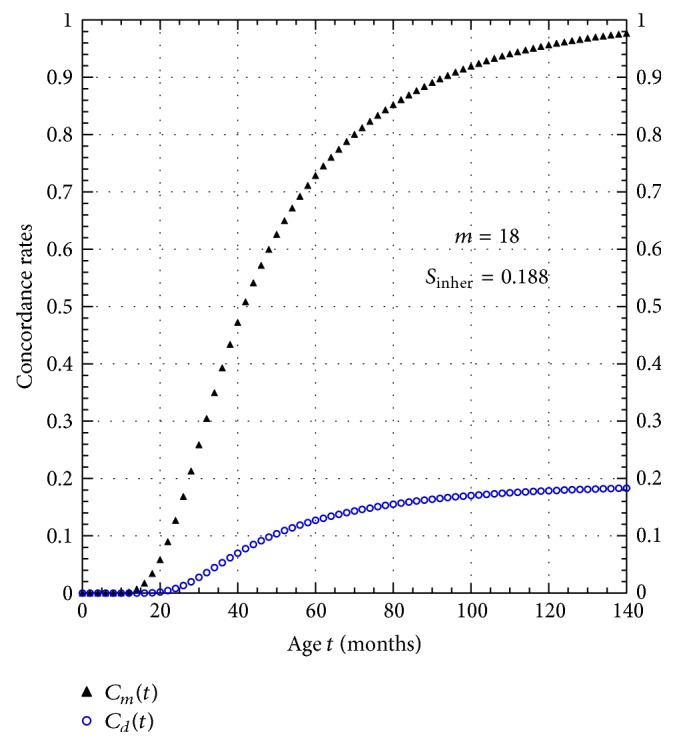
Plot of monozygote *C*_*M*_(*t*) and dizygote *C*_*D*_(*t*) concordance as functions of cohort age *t* obtained from model simulation of ASD data.

The actual monozygote concordance curve *C*_*m*_(*t*) predicted by the model fit to IAN data (see (B.4a)) is plotted in Figure 4. From the concordance curve *C*_*m*_(*t*) we see that 50% of the monozygote twin pairs become concordant at the age of 42 months while 96% concordance is reached by the age of 123 months (10.25 years). This latter result suggests the easiest way of testing the veracity of the multimutation model of ASD development. The model predicts that the monozygote concordance rate curve will be a monotonically increasing function of age that will eventually saturate at 100%. By following a monozygote twin cohort as it ages to a point where all members of the cohort are, say, over the age of 10 years of age, we can easily test the model prediction of 100% saturation in the concordance curve *C*_*m*_(*t*).

We will now simulate the results obtained from the 2014 IAN data by assuming that these results were obtained by a postulated cohort whose members all have the same age *t* (an* age-cohort* simulation). In this simulation, the observed value of the monozygote concordance rate of 41/60 (68.3%) is set equal to *C*_*M*_(*t*) and the observed value of the dizygote concordance rate of 49/260 (18.8%) is set equal to *C*_*D*_(*t*). In the age-cohort simulation, (B.4b) gives *P*_*s*_(*t*) = 82/101 = 0.812, from which we find that the age of the cohort when *C*_*M*_(*t*) = 41/60 is *t* = 55 months (4.58 years), significantly* higher *than the* average* age of 41.2 months for the 2014 IAN cohort. This last result is completely reasonable and follows from the nonlinear nature of the *P*_*s*_(*t*) function shown in Figure 2(a). From (B.6c), the age-cohort simulation gives *S*_inher_ = 0.188 (18.8%), so that the probability that the cotwin of the index twin will also turn out to develop ASD is 18.8%. Using this value for *S*_inher_ in (B.6a) gives the dizygote concordance curve *C*_*D*_(*t*), which is also plotted in Figure 4. Notice that the dizygote concordance curve characteristically saturates at the value of *S*_inher_ (18.8% here), but, by contrast, the monozygote concordance curve characteristically saturates at 100%.

For an ASD susceptible cohort of monozygote twins whose members all have the same age *t*, the monozygote concordance *C*_*M*_(*t*) is given by (B.4a). If the members of the susceptible cohort have different ages, we need to derive a result for the* expected* concordance for the diverse cohort. Suppose that *n*_*i*_ members of the susceptible cohort have the same age *t*_*i*_, where *i* = 1, 2, 3,…, and the total number of members of the cohort is *N*_*T*_ = *n*_1_(*t*_1_) + *n*_2_(*t*_2_) + *n*_3_(*t*_3_)+⋯. Then, the expected value of the concordance for the entire cohort is coincident with its* average* value, namely,

(2) $$\begin{array}{c} \langle C_{M}\rangle =\frac{\sum_{i=1,2,\ldots }C_{M}\left(t_{i}\right)\cdot n_{i}\left(t_{i}\right)}{N_{T}}. \end{array}$$

For this expression to yield an accurate value, the values of *n*_*i*_ must be great enough so that these members of the cohort are described by the age-of-onset age distribution of the cohort as a whole. If *N*_*T*_ is large enough, we can set

(3a) $$\begin{array}{c} n_{i}⟹dn\equiv N_{T}dP_{n}\left(t\right), \end{array}$$

provided *t*_*i*_ = *t* and the function *P*_*n*_(*t*) is defined as the cumulative probability that a member of the cohort will have an age *t* or less. If the youngest member of the cohort is *t*_*y*_ and the oldest member of the cohort is *t*_*o*_ then (2) gets replaced by

(3b) $$\begin{array}{c} \langle C_{M}\rangle =\overset{t_{o}}{\underset{t_{y}}{\int }}C_{M}\left(t\right)\cdot dP_{n}\left(t\right). \end{array}$$

The result in (3b) assumes that the ages of the members of the cohort can be approximated by the continuous probability distribution *P*_*n*_(*t*).

As an example of the use of (3b), let us assume that *P*_*n*_(*t*) = *P*_*s*_(*t*) itself. What situation would this describe? Suppose we randomly divide up the monozygote pairs of a cohort susceptible to developing ASD by putting one twin into group A and the other twin into group B. Each of these two groups will be described by the same age-of-onset distribution *P*_*s*_(*t*). Suppose we focus entirely on the members of group A. As the members of group A develop ASD, *dP*_*n*_(*t*) = *dP*_*s*_(*t*) and we can now go ahead and evaluate (3b). Assuming that the ages in the cohort ranges from a low of *t*_*y*_ = 0 to a high of *t*_*o*_, plugging (B.4a) into (3b) and integrating gives

(4a) $$\begin{array}{c} \langle C_{M}\left(t\right)\rangle =-1-\frac{2}{P_{s}\left(t_{o}\right)}\ln\left[1-\frac{P_{s}\left(t_{o}\right)}{2}\right], \end{array}$$

where “ln” is the natural logarithm function. If *t*_*o*_ = *∞*, then since *P*_*s*_(*∞*) = 1, (4a) gives

(4b) $$\begin{array}{c} \langle C_{M}\rangle =2\ln\left(2\right)-1=0.38629. \end{array}$$

Perhaps the most amazing aspect of the result in (4b) is that it is completely independent of the exact expression for *P*_*s*_(*t*) since (4b) only depends on the characteristic features of *P*_*s*_(*t*), namely, that it is continuous and obeys *P*_*s*_(0) = 0 and *P*_*s*_(*∞*) = 1. Of course, the result in (4b) also depends on the model that led to the result in (B.4a) in Appendix B, including the assumption that identical twins are equally likely to develop ASD. The result in (4b), namely, that the average concordance for this age distribution is 38.6%, probably represents the* minimum* value to be expected for the monozygote concordance because the average age of cohorts used in twin studies are generally higher than the average age-of-onset (3.52 years) for the cohort used to compute the result in (4b). It is important to emphasize that the result in (4b) can easily be tested by using ASD data for any known cohort.

The measured ASD monozygote concordance rate using the IAN database varies from 41/60 = 0.683 (2014) to 59/67 = 0.881[2] or somewhere between 68.3% and 88.1%. What does the multimutation model predict the monozygote concordance rate to be?

Using (2) with each *n*_*i*_(*t*_*i*_) = 1, gives an average concordance of 〈*C*_*M*_〉 = 0.755 (75.5%). Because the sample was so small (all the *n*_*i*_(*t*_*i*_) = 1), we would not expect exact agreement with observation using (2) in this case. Still, the model prediction for the* average* monozygote concordance using (2) (75.5%) is within the observed range for the overall rate (68.3%–88.1%) for the IAN database, lending credence to the multimutation model.

For the dizygote twins in this IAN cohort we found a concordance rate of 49/260 = 0.188 (18.8%). Since the observed value of the dizygote concordance typically varies between 10–30%,* below* the expected* minimum *monozygote concordance rate of 38.6% (see (4b)), the multimutation model of ASD development passes another critical test.

From Figure 2(a) notice that if *t*_*y*_ ≥ 120 months (10 years), then from (3b), 〈*C*_*M*_(*t*)〉 → 1. Thus, the only conclusive test of the genetic multimutation model is to repeatedly measure the concordance of any cohort as it ages until its youngest member is older than 10 years of age since this model predicts that the concordance will approach 100% under these conditions.

A very similar study of ASD twin concordance in 192 twins born between 1987 and 2004 was conducted by Hallmayer et al. using California Department of Developmental Services data [7]. This study found an overall ASD monozygote concordance rate of 32/54 = 0.5926 (59.2%) and a dizygote concordance rate of 18/138 = 0.1304 (13.0%), rates that are both moderately lower than the respective rates obtained in our current study. However, before any conclusion can be drawn from the fact that the monozygote concordance in this study (59.2%) is significantly lower than that of our current study (69.0%), for example, the ASD age of first diagnosis and age distribution curves of the Hallmayer et al. cohort must be known, and* these data are not provided in*  [7]. The only information we have about the twin pairs in Hallmeyer study are their mean ages: 13.44 years for the monozygote twins and 12.01 years for the dizygote twins. Notice that the value of the concordance integral in (3b) depends crucially on the age of first diagnosis and cohort age distribution curves; thus, the possibility that the difference in the mean monozygote concordance rate between our IAN study and that of Hallmayer is entirely due to a difference between these two respective distribution curves must now be explored.

**Figure 5:**
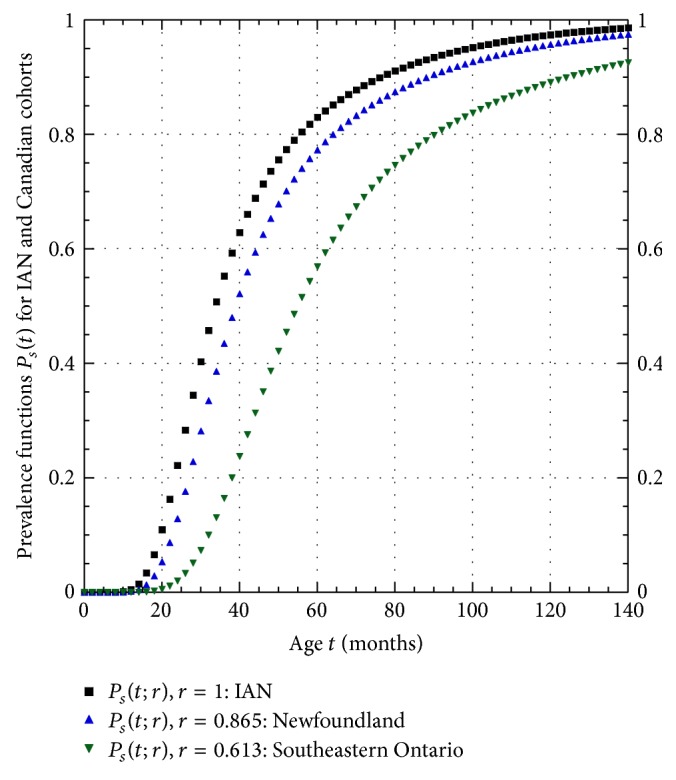
ASD age-of-onset prevalence function *P*_*s*_(*t*) for IAN, Newfoundland, and Southeastern Ontario cohorts.

An analysis of ASD age of first diagnosis distribution curves in four different regions of Canada demonstrated that the distribution curves were all significantly different from each other [8]. The median age children were first diagnosed with ASD varied from a low of 39.0 months in Newfoundland and Labrador to 55.0 months in Southeastern Ontario. Using the form of the model prevalence function in (1), we generalize this function to give a prevalence function now given by

(5) $$\begin{array}{c} P_{s}\left(t;r\right)={\left[1-\exp\left[-k_{1}\left(r\cdot t\right)\right]\right]}^{m_{1}}\left[1-\exp\left[-k_{2}\left(r\cdot t\right)\right]\right], \end{array}$$

where the dimensionless scaling factor *r* can be viewed as slowing down (*r* < 1) or speeding up (*r* > 1) the rate at which ASD mutations occur; for these reasons, the parameter *r* will be called the* biological clock rate*. The values of *m*_1_, *k*_1_, and *k*_2_ in (5) remain the same as they are in Table 1 for the IAN cohort, but we will let the value of the biological clock rate parameter *r* float to fit the Canadian data. Setting *P*_*s*_(39*m*) = *P*_*s*_(55*m*) = 1/2, we get the following two extreme values for the biological mutation rate: *r*_39_ = 0.865 and *r*_55_ = 0.613, both lower than the value of *r* = 1 that fits the IAN data. Plots of these 3 different age-of-onset prevalence functions appear in Figure 5. The ratio *r*_55_/*r*_39_ = 0.720 indicates that there is significant variation in the mutation rate from region to region in Canada, and the same may be true in the USA. The fact that *r*_55_ < *r*_39_ indicates that the age-of-onset curve in Southeastern Ontario grows more slowly than the age-of-onset curve in Newfoundland, as shown in Figure 5. This in turn would mean that the ASD monozygote concordance rate for a cohort whose members have the same age *t* in Southeastern Ontario would be less than the rate for an identical cohort at the same age in Newfoundland. Using these results for Canada as a guide, the disparity in monozygote concordance rates between the Hallmayer et al. [7] and IAN studies (59.1% versus 68.3%, resp.) may be entirely due to the value of biological clock rate parameter in the Hallmaker study being *r*_*H*_ < 1 (see Figure 5 for two *r* < 1 examples).

Thus, it is possible to reproduce the Hallmayer et al. results using the same multimutation model (MMM) that fit our IAN analysis by simply changing the value of a single parameter in our MMM model and using a different cohort age distribution curve. Since the ASD age of first onset and age distribution curves for the Hallmayer et al. [7] cohort are missing from this paper,* no model analysis of this data is possible.* However, the success of the multimutation model analysis presented here suggests that ASD may be a purely genetically driven disease. The only way to conclusively test the genetic model of ASD development is to conduct follow-up studies of the original cohort to test the model's predictions, namely, that as a cohort of twins ages, the monozygote concordance rate will approach 100% while the dizygote concordance rate will saturate at a value less than 38.6% (see (4b)).

## 3. Discussion

The multimutation model of ASD development constructed here has been shown to agree with a wide variety of age-of-onset data. The model assumes that susceptibility to develop the disease is present at birth (perhaps acquired at conception) and that the full manifestations of the disease occurs when all of the specific mutations of the brain characteristic of ASD are acquired. If the necessary number of characteristic mutations needed to cause ASD occurs at an age after the child learns how to talk, the verbal abilities of the child will regress, leading to the term “late” ASD. However, our model makes no distinction between “early” or “late” ASD since the underlying mechanism leading to the development of this disease is independent of the age-of-onset. In the model presented here these mutations can be acquired in any order, but a model in which these mutations can only occur in a definite order yields very similar results.

Support for this theoretical model comes from a recent controlled study of postmortem brain scans of children with ASD by Stoner et al. [9]. This study examined the brain tissue of 22 children who died between the ages of 2 and 15, half of whom had autism. In 10 out of the 11 autistic brains the researches found abnormalities in patches of the temporal and prefrontal cortex, areas of the brain associated with language and cognition, which could be responsible for some of the symptoms of autism.

If ASD is a purely genetically driven disease, then if one identical twin develops the disease, the companion twin must develop it as well. There is an easy way to test for this possibility, namely, continue to monitor the companion twin as he or she ages. From the age-of-onset prevalence curve in Figure 2(a) it is clear that over 98% of those susceptible to developing ASD acquire it by the age of 124 months. Therefore, from Figure 4 we see that we would expect over 96% concordance in identical twin pairs by this age. Therefore, all we need to do to test the genetic hypothesis of ASD development is to continue to survey the concordance rate in susceptible twin pairs until they all exceed, say, 10 years of age.

## Figures and Tables

**Table 1:** Values of intrinsic ASD and schizophrenia model parameters stemming from fits to age-of-onset data.

Cohort and gender	*k*_1_ (per month)	*k*_2_ (per month)	*m* = *m*_1_ + 1
ASD males (M)	0.12605 m^−1^	0.030207 m^−1^	18
ASD females (F)	0.12532 m^−1^	0.031716 m^−1^	18
Schizophrenia (M + F)	0.10757 y^−1^	0.029959 y^−1^	16

## Appendices

### A. The* Independent Mutation* Model of Autism Spectrum Disorder Development for Singleton Births

Suppose autism spectrum disorder (ASD) requires a set of *m* random mutations within the brain that can occur independently of one another, where *m* = 1,2, 3,…. Accordingly, this model will be called the* independent* multimutation model (MMM).

Consider a random sample of a population whose members were all born on the same date and who are all susceptible to developing ASD. It will be assumed that a total of *m* specific, special mutations of the brain is necessary to cause the disorder. The size of the susceptible population will be denoted by *N*_*s*_ and the cumulative number of people in this sample that has developed autism at age *t* will again be denoted by *N*_*m*_(*t*). The fraction of the population that has not developed the *i*th mutation at age *t*, where *i* = 1,2,…, *m*, is given by exp⁡(−*k*_*i*_ *t*), where *k*_*i*_ is defined as the *i*th mutation rate (a constant). The meaning of “mutation” here generically refers to any internally driven biological change that contributes to the onset of ASD and does not necessarily refer to a change in genomic sequence. Thus, the fraction of the susceptible population that has developed the *i*th mutation at age *t*, equivalent to the probability *p*_*i*_(*t*) of developing this mutation by age *t*, is given by

(A.1) $$\begin{array}{c} p_{i}\left(t\right)\equiv 1-\exp\left(-k_{i}t\right),\;i=1,2,3,\ldots ,m, \end{array}$$

where the mutation rate *k*_*i*_ is related to the average time *T*_*i*_ necessary for this mutation to occur through *T*_*i*_ = 1/*k*_*i*_. If *m* independent mutations are required for ASD to develop, then the probability that the disorder will develop at age *t* in the susceptible population, a quantity to be called the susceptible prevalence, is given by

(A.2) $$\begin{array}{c} P_{s}\left(t\right)=\frac{N_{m}\left(t\right)}{N_{s}}=p_{1}\left(t\right)p_{2}\left(t\right)p_{3}\left(t\right)\cdots p_{m-1}\left(t\right)p_{m}\left(t\right), \end{array}$$

where the values of the *m* mutation rates (constants) are generally all independent of each other. Thus, in this model the mutations can occur in any order, simultaneously, or at completely different times. Notice that the maximum possible value of *P*_*s*_(*t*) is 1.

Now suppose a total of *N*_*b*_ infants are born on a given date in a given country. If a fraction *f*_*s*_ of this infant population is susceptible to developing ASD, then the number of people in this cohort that is susceptible to developing the disorder is given by *N*_*s*_ = *f*_*s*_ *N*_*b*_. Let the cumulative number of the *N*_*b*_ infants that has developed ASD by age *t* be denoted by *N*(*t*).

Then, the prevalence or risk of ASD for the entire population is given by

(A.3) Pt=NtNb=fsPst=fs·p1tp2tp3t⋯pm−1tpmt,              fs=NsNb.

The fraction of the population that develops ASD between the ages of *t* and *t* + *dt* is given by *dP*(*t*), so that the fractional incidence rate is given by

(A.4) $$\begin{array}{c} \text{IR}\left(t\right)=\frac{dP\left(t\right)}{dt}=f_{s}\frac{dP_{s}\left(t\right)}{dt}\equiv f_{s}\cdot {\text{IR}}_{s}\left(t\right). \end{array}$$

The best fits to ASD data occurred if *m*_1_ = *m* − 1 mutation rates are all equal to the same constant rate *k*_1_ while the remaining one is equal to another rate *k*_2_ ≠ *k*_1_; then, the prevalence function in (A.3) becomes

(A.5) Pt=NtNb=fsPst=fs·1−exp⁡⁡−k1tm11−exp⁡−k2t,              fs=NsNb,

where *m* = *m*_1_ + 1. Notice that this model requires determining the values of 4 parameters (*f*_*s*_, *k*_1_, *k*_2_, and *m*). If it turns out that *k*_2_ = *k*_1_, then the number of parameters in (A.5) is reduced to 3, which is the simplest possible model.

Remarkably, the prevalence function shown in (A.5) also gave the best fit to schizophrenia age-of-onset data with different values for the intrinsic parameters *k*_1_, *k*_2_, and *m*, as seen in Table 1 [1]. Notice that the total number of characteristic mutations necessary to cause ASD and schizophrenia are comparable, but the values of the mutation rates *k*_1_ and *k*_2_ for these two disorders have completely different scales (months versus years). The fact that the age-of-onsets distributions for these two disorders are fit by the same function shown in (A.5) supports the possibility that there is a connection between ASD and schizophrenia, an idea that has been extensively discussed in the literature (see e.g., [10]). Clearly, these disorders have many clinical features in common.

The values of the parameters in the prevalence function in (A.5) or its corresponding incidence function, depend on the values of four fit parameters, *f*_*s*_, *k*_1_, *k*_2_, and *m*_1_ (or, equivalently, *m*), whose values are determined by a least-squares fit to appropriate data.

If the set of *n* consecutive data-values used in the fit are denoted by {*d*_*i*_}, and if the corresponding model fit-values are denoted by {*x*_*i*_}, then the square of the error of the fit, to be called *χ* ^2^ (chisquare), is defined as

(A.6) $$\begin{array}{c} \chi^{2}\equiv \overset{n}{\underset{i=1}{\sum }}{\left[x_{i}-d_{i}\right]}^{2}. \end{array}$$

The lower the value of chisq, the better the model fit's the data.

Now the average ASD age of onset $\overset{-}{t}$ of a random cohort of patients in the MMM is defined by

(A.7) $$\begin{array}{c} \overset{-}{t}\equiv \langle t\rangle \equiv \overset{t=\infty }{\underset{t=0}{\int }}t\;dP_{s}\left(t\right), \end{array}$$

where *P*_*s*_(*t*) is given in (A.5).

The standard deviation *t*_*sd*⁡_ from the mean $\overset{-}{t}$ is defined through

(A.8) $$\begin{array}{c} t_{\mathrm{sd}}^{2}\equiv \overset{t=\infty }{\underset{t=0}{\int }}{\left(t-\overset{-}{t}\right)}^{2}dP_{s}\left(t\right)=\overset{\infty }{\underset{0}{\int }}t^{2}dP_{s}\left(t\right)-{\overset{-}{t}}^{2}=\langle t^{2}\rangle -{\langle t\rangle }^{2}. \end{array}$$

Clearly both $\overset{-}{t}$ and *t*_*sd*⁡_ are functions of the model parameters *k*_1_, *k*_2_, and *m*_1_ = *m* − 1.

### B. Modeling ASD Twin Study Data

The multimutation model (MMM) of ASD development constructed in Appendix A for singleton births will now be extended to describe twin births. In a collection of monozygote twins where one of the twins has developed ASD, every member of the cohort is born with a susceptibility to develop the disorder, and so the risk fraction or lifetime ASD risk is *f*_*s*_ = 1. In the analysis of all such studies, each twin in a given pair must be randomly assigned to two different sub-cohorts using a criterion that has nothing to do with the disorder, for example, by the random flipping of a coin. Thus, the genetic profile of the two randomly constructed subcohorts are identical. If the development of ASD is entirely driven by genetics, with environmental factors having nothing to do with it, then the age-of-onset prevalence curve *P*_*s*_(*t*) of the two subcohorts should be identical even though this function may have a form very different from (A.5) in Appendix A. This prediction is one of the critical tests of the genetic model of ASD development, and it is essential that published ASD twin studies be brought up-to-date in order to test this model.

In our ASD model, all members of both subcohorts are born with the susceptibility to develop the disorder. Thus, the genetic model predicts that all monozygote cotwins will eventually develop ASD.

When one twin (the* index* twin) in each pair has developed the disorder, the other twin will be referred to as the* cotwin* in this paper. Our model posits that in monozygote twin pairs the co-twin has the same susceptibility to develop ASD as the index twin. Consider a cohort of monozygote twins all having the same age. Assuming birth is coincident with age *t* = 0, one twin (either the first or second born) will experience the onset of ASD first, say at age *t*. As soon as that happens, one twin is randomly assigned to subcohort 1 and the other to subcohort 2. All ASD twin studies can easily assemble subcohorts 1 and 2 in this way. As these subcohorts age, their cotwins start experiencing the onset of the disorder leading to concordance. The risk of developing ASD at age *t* by members of a subcohort is given by a* susceptible* prevalence function *P*_*s*_(*t*) which in our modeling is defined in (A.5). Assuming that genetic factors are entirely responsible for the development of the disorder, for any cohort of monozygote twins, both susceptible subcohorts will experience the same susceptible prevalence function *P*_*s*_(*t*).

In general, the probability that a member of subcohort 1 will have developed (will not have developed) ASD by age *t* will be denoted by *P*_*s*_^(1)^(*t*) (*Q*_*x*_^(1)^(*t*) ≡ 1 − *P*_*s*_^(1)^(*t*)), with a similar notation for subcohort 2. Since *P*_*s*_^(1)^(*t*) + *Q*_*x*_^(1)^(*t*) = 1 for *i* = 1,2, we have

(B.1a) 1=Ps1t+Qx1tPs2t+Qx2t=Ps1(t)Ps2(t)+Ps1tQx2t+Qx1tPs2t +Qx1tQx2t.

Thus, we define subcohort concordance, discordance, and nondisease probabilities as

(B.1b) Ps,s(t)=Ps(1)(t)Ps(2)(t), Ps,x(t)=Ps(1)(t)Qx(2)(t)+Qx(1)(t)Ps(2)(t), Px,x(t)=Qx(1)(t)Qx(2)(t),

respectively, where

(B.1c) $$\begin{array}{c} P_{s,s}\left(t\right)+P_{s,x}\left(t\right)+P_{x,x}\left(t\right)=1. \end{array}$$

It is important to note that subcohort concordance as defined above, for example, is not the same as pairwise concordance as usually defined in the literature. Here, if a member of subcohort 1 and a member of subcohort 2 are chosen at random at age *t*, the probability that* both* will have acquired ASD is given by *P*_*s*,*s*_(*t*), and the probability that they will be found to be discordant is *P*_*s*,*x*_(*t*); the probability that neither will be found to have ASD at age *t* even though they are both susceptible to developing the disorder is *P*_*x*,*x*_(*t*).

For monozygote [MZ] twins, subcohorts 1 and 2 are genetically identical so that *P*_*s*_^(1)^(*t*) = *P*_*s*_^(2)^(*t*) ≡ *P*_*s*_(*t*) and *Q*_*x*_^(1)^(*t*) = *Q*_*x*_^(2)^(*t*) ≡ *Q*_*x*_(*t*). Thus, the probabilities in (B.1b) become

(B.2) Ps,st=Ps2t,Ps,xt=2PstQxt,Px,xt=Qx2t,iiiMZ  twins.

When a susceptible monozygote twin pair in the (*x*, *x*) state (neither twin has developed ASD yet) makes a transition to the (*s*, *x*) state at age *t*, it means that one of the twins has developed the disorder at age *t* (the age-of-onset). The probability that such a transition would take place between the ages of *t* and *t* + *dt*, denoted by *dP*_*s*,*x*_^(+)^(*t*), is given by

(B.3a) $$\begin{array}{c} dP_{s,x}^{\left(+\right)}\left(t\right)=-dP_{x,x}\left(t\right). \end{array}$$

Integrating this result from age *t* = 0 to any age *t* gives

(B.3b) $$\begin{array}{c} P_{s,x}^{\left(+\right)}\left(t\right)=1-P_{x,x}\left(t\right)=1-{\left[1-P_{s}(t)\right]}^{2}=P_{s}\left(t\right)\left[2-P_{s}\left(t\right)\right] \end{array}$$

since *P*_*s*,*x*_^+^(0) = 0 and *P*_*x*,*x*_(0) = 1. Since *P*_*s*,*x*_^(+)^(*t*) is the age-of-onset distribution curve for the first twin of a pair that is susceptible to developing ASD, the result in (B.3b) is extremely important in describing monozygote twin discordance. It is also important to point out that the prevalence function *P*_*s*_(*t*) appearing in all the equations in Appendix B is completely independent of the mutation model version of this function constructed in Appendix A.

In ASD twin studies, the cohort consists of only the concordant and discordant twin cases since, to date, it remains difficult to determine susceptibility to ASD unless the disorder actually develops. Thus, referring back to the results in (B.2) in Appendix B, the fraction of the monozygote cohort that is concordant at age *t* at the end of a given twin study, denoted by *C*_*M*_(*t*), is given by

(B.4a) CMt≡Ps,stPs,st+Ps,xt=Ps2tPs2t+2PstQxtCMt=Ps(t)Ps(t)+21−Ps(t) or CMt≡Ps,stPs,st+Ps,xt=Pst2−Pst.

Notice that the monozygote concordance rate *C*_*M*_(*t*) is a function of *P*_*s*_(*t*).

Since *P*_*s*_(0) = 0 and *P*_*s*_(*∞*) = 1, the monozygote concordance rate also varies between 0 and 1. Inverting (B.4a) by solving for *P*_*s*_(*t*) gives

(B.4b) $$\begin{array}{c} P_{s}\left(t\right)=\frac{2C_{M}\left(t\right)}{\left[1+C_{M}\left(t\right)\right]},\;\left[\text{MZ}\;\text{twins}\right]. \end{array}$$

Since the value of *C*_*M*_(*t*) is determined from twin studies, the result in (B.4b) is a model prediction of the value of *P*_*s*_(*t*); this prediction can be tested by reanalyzing the data in twin studies to compute this quantity.

For the dizygote twin cases the formal results in (B.1a), (B.1b), and (B.1c) in Appendix B carry over here. Keeping the subscript (1) to refer to the autistic index twin and subscript (2) to refer to the* fraternal* cotwin, a new expression for *C*_*M*_(*t*) must be developed. To this end, we define the probability that a fraternal cotwin of the index twin will also inherit the susceptibility to develop ASD and denote this probability by *S*_inher_. Then, we can set

(B.5a) Ps2(t)=SinherPs1(t)≡SinherPs(t) withQx(2)(t)≡1−Ps(2)(t)

as usual. Using (B.5a) in (B.1a), (B.1b), and (B.1c) then gives, analogous to (B.2),

(B.5b) Ps,st=SinherPs2t,

(B.5c) Ps,xt=Pst1−SinherPst+1−PstSinherPstPs,xt=1+SinherPs(t)−2SinherPs2(t)

for dizygote twins.

In the same way, the fraction of the dizygote cohort that is concordant at the age *t* at the end of the study, denoted by *C*_*D*_(*t*), is given by

(B.6a) $$\begin{array}{c} C_{D}\left(t\right)\equiv \frac{P_{s,s}\left(t\right)}{\left[P_{s,s}\left(t\right)+P_{s,x}\left(t\right)\right]}=\frac{S_{\text{inher}}P_{s}\left(t\right)}{\left[1+S_{\text{inher}}-S_{\text{inher}}P_{s}\left(t\right)\right]}. \end{array}$$

Notice that the dizygote concordance rate *C*_*D*_(*t*) is also a function of *P*_*s*_(*t*). Since *P*_*s*_(0) = 0 and *P*_*s*_(*∞*) = 1, the monozygote concordance rate also varies between 0 and *S*_inher_. Solving (B.6a) for the unknown probability *S*_inher_ gives

(B.6b) $$\begin{array}{c} S_{\text{inher}}=\frac{C_{D}\left(t\right)}{\left[P_{s}\left(t\right)+\left\{P_{s}\left(t\right)-1\right\}C_{D}\left(t\right)\right]}. \end{array}$$

Using (B.4b) in (B.6b) gives

(B.6c) $$\begin{array}{c} S_{\text{inher}}=\frac{\left[1+C_{M}\left(t\right)\right]}{\left[2C_{M}\left(t\right)/C_{D}\left(t\right)-1+C_{M}\left(t\right)\right]}. \end{array}$$

The values of the monozygote and dizygote concordance fractions *C*_*M*_(*t*) and *C*_*D*_(*t*), respectively, are determined by twin studies, so (B.6c) is a model prediction of the value of *S*_inher_, the probability that a fraternal cotwin of an index twin will also inherit the susceptibility to develop ASD. This prediction of the model can be tested by reanalyzing the data in classical twin studies to compute the value of *S*_inher_. It is again important to note that the prevalence function *P*_*s*_(*t*) in Appendix B is independent of any mutation model version of this function; thus, all the formulas from (B.1a) to (B.6c) in Appendix B *are independent of any model*.
